# Antibacterial and Bactericidal Effects of the Er: YAG Laser on Oral Bacteria: A Systematic Review of Microbiological Evidence

**DOI:** 10.3390/jfb16060209

**Published:** 2025-06-03

**Authors:** Jakub Fiegler-Rudol, Dariusz Skaba, Aleksandra Kawczyk-Krupka, Rafał Wiench

**Affiliations:** 1 Department of Periodontal Diseases and Oral Mucosa Diseases, Faculty of Medical Sciences in Zabrze, Medical University of Silesia, 40-055 Katowice, Poland; rwiench@sum.edu.pl; 2 Department of Internal Diseases, Angiology and Physical Medicine, Centre for Laser Diagnostics and Therapy, Medical University of Silesia in Katowice, Batorego 15, 41-902 Bytom, Poland; akawczyk@sum.edu.pl

**Keywords:** Er:YAG laser, antimicrobial efficacy, dental disinfection, biofilm removal, in vitro study, laser therapy, endodontics, periodontology

## Abstract

Background: The Er:YAG laser has gained attention in dentistry for its potential to enhance microbial disinfection through targeted photothermal and photoacoustic mechanisms. Objective: This systematic review aimed to evaluate the antibacterial and bactericidal efficacy of Er:YAG laser therapy across clinically relevant oral pathogens in in vitro models. Methods: Following the PRISMA 2020 guidelines, a systematic search of PubMed, Embase, Scopus, and the Cochrane Library was conducted for studies published between 2015 and 2025. The review protocol was registered with PROSPERO (CRD420251031368). Eligibility criteria included in vitro or animal studies assessing the bactericidal effects of the Er:YAG laser on oral bacteria or fungi, either alone or in combination with chemical disinfectants. Study selection, data extraction, and quality assessment were conducted independently by multiple reviewers. Results: Ten in vitro studies met inclusion criteria. The Er:YAG laser demonstrated significant antibacterial effects against *Enterococcus faecalis*, *Streptococcus mutans*, *Porphyromonas gingivalis*, *Candida albicans*, and other species. Greater bacterial reduction was consistently observed when the laser was combined with adjunctive irrigants such as sodium hypochlorite or hydrogen peroxide. The laser was effective in reducing biofilm biomass and viable counts, particularly in complex anatomical settings. Most studies were rated as low risk of bias. Conclusions: Er:YAG laser therapy is a promising adjunctive tool for microbial disinfection in dentistry, particularly in challenging anatomical sites. Further well-designed in vivo and clinical studies are needed to confirm its efficacy and determine optimal treatment parameters.

## 1. Introduction

Lasers have become increasingly prominent in modern dental practice, where innovative technologies are continually sought to enhance both clinical outcomes and patient comfort [1,2,3,4,5,6]. Among these technologies, the Erbium-doped Yttrium–Aluminum–Garnet (Er:YAG) laser has drawn attention due to its unique photothermal properties, which enable simultaneous ablation of dental hard tissues and microbial reduction [7,8]. Unlike conventional approaches that rely heavily on chemical irrigants or mechanical instrumentation for disinfection, the Er:YAG laser delivers energy precisely to targeted sites, potentially improving penetration into complex anatomical areas such as dentinal tubules or periodontal pockets [9,10]. This targeted energy release may offer improved bactericidal effects, thereby reducing the likelihood of persistent or recurrent infections [7,8,9,10,11].

Despite the proven efficacy of traditional non-laser alternatives, such as sodium hypochlorite, chlorhexidine, or hydrogen peroxide, their limitations have become more evident in recent years [12,13,14,15,16]. Chemical irrigants, for instance, can pose significant drawbacks including unpleasant taste, cytotoxicity to oral tissues if overextended, and potential allergies or sensitivities in some patients [17]. Furthermore, the stability and activity of these chemicals can be adversely affected by organic matter or certain pH conditions, ultimately undermining their antimicrobial performance [17]. However, their limitations have become more apparent in recent years [15,16]. These include unpleasant taste, cytotoxicity when extruded beyond the apex, and potential allergic reactions or sensitivities in susceptible individuals [17]. Moreover, their antimicrobial effectiveness may be compromised by interactions with organic matter, suboptimal pH conditions, or dentin buffering, which can deactivate active components [17]. Mechanical instrumentation, although indispensable, also has inherent limits; endodontic files and manual scraping do not always reach the intricate branching of root canal systems or the more inaccessible areas of subgingival biofilms [17,18,19]. This leaves behind microbial reservoirs that can perpetuate infection and compromise long-term treatment success [15,16,17,18,19,20]. In response to these concerns, the Er:YAG laser has been proposed as an adjunct or alternative that might circumvent some of these shortcomings [9,20,21,22]. Its ability to create micro-explosions of steam within hard and soft tissues not only disrupts resistant biofilms but also preserves healthy tissue integrity [23]. In response, various laser systems have been introduced to augment decontamination efforts. Diode lasers (810–980 nm) are commonly used for soft tissue decontamination and are effective against pigmented bacteria due to their absorption by melanin and hemoglobin, though they lack ablative capability on hard tissues [21]. Nd:YAG lasers (1064 nm) offer deeper penetration into soft tissues and have demonstrated bactericidal effects, particularly in periodontal applications [22]. CO_2_ lasers (10,600 nm) provide strong soft tissue ablation and hemostasis but are less commonly used for deep bacterial elimination due to their superficial absorption profile [23]. Er:YAG lasers, operating at 2940 nm, exhibit high absorption in water and hydroxyapatite, making them uniquely suited for both hard and soft tissue applications, including smear layer removal and biofilm disruption [24].

The Er:YAG laser eliminates bacteria through a combination of photothermal and photomechanical effects [24]. Its 2940 nm wavelength is highly absorbed by water and hydroxyapatite, allowing for efficient energy transfer to both the irrigant and dentin [25]. This interaction generates cavitation bubbles and shock waves within the irrigant, a phenomenon known as photoacoustic streaming, which enhances the fluid’s penetration into complex canal anatomies and dentinal tubules [26]. Additionally, the laser’s ability to ablate the smear layer exposes dentinal tubule openings, facilitating deeper disinfectant infiltration [27]. The resulting high-energy microenvironment disrupts bacterial biofilms, damages cell walls, and improves the bactericidal effectiveness of irrigants like sodium hypochlorite [28]. Collectively, these effects contribute to the Er:YAG laser’s potent antimicrobial action, especially in areas inaccessible to traditional instruments.

However, questions remain about the generalizability of these observed antimicrobial effects, given the variety of laser parameters, bacterial strains, and study designs reported in the literature [16,17,18,19,20]. Therefore, a systematic evaluation of these studies is vital to clarify the precise bactericidal capabilities of Er:YAG lasers and to determine how factors such as energy output, wavelength, exposure time, and adjunctive chemical agents might optimize clinical outcomes.

This review is novel in its exclusive focus on in vitro microbiological outcomes related to Er:YAG laser use in dentistry. This review aims to synthesize the current evidence on the antibacterial efficacy of Er:YAG lasers in dentistry. By collating and critically evaluating findings from diverse experimental protocols, we seek to identify both the strengths of Er:YAG therapy and the gaps in our understanding. Ultimately, the insights gained here will help inform clinical decisions and guide future research into refining laser-based approaches for effective infection control. While previous reviews have addressed general clinical applications of lasers in dentistry, our review is distinct in its exclusive focus on microbiological outcomes derived from in vitro studies using the Er:YAG laser. We systematically evaluate its antibacterial and bactericidal performance across diverse pathogens, surface types, and anatomical models, with a dedicated quality assessment framework adapted to laser-based microbiological research. This approach provides a more mechanistic and pathogen-specific synthesis of the evidence, which has been lacking in the prior literature.

## 2. Materials and Methods

### 2.1. Focused Question

A systematic review was conducted using the PICO framework [29], structured as follows: In patients with microbial infections (Population), does treatment with the Er:YAG laser (Intervention), compared to conventional antimicrobial therapies, alternative laser modalities, or no laser treatment (Comparison), lead to superior bacterial eradication or reduction in microbial load (Outcome)?

### 2.2. Search Strategy

**Figure 1:**
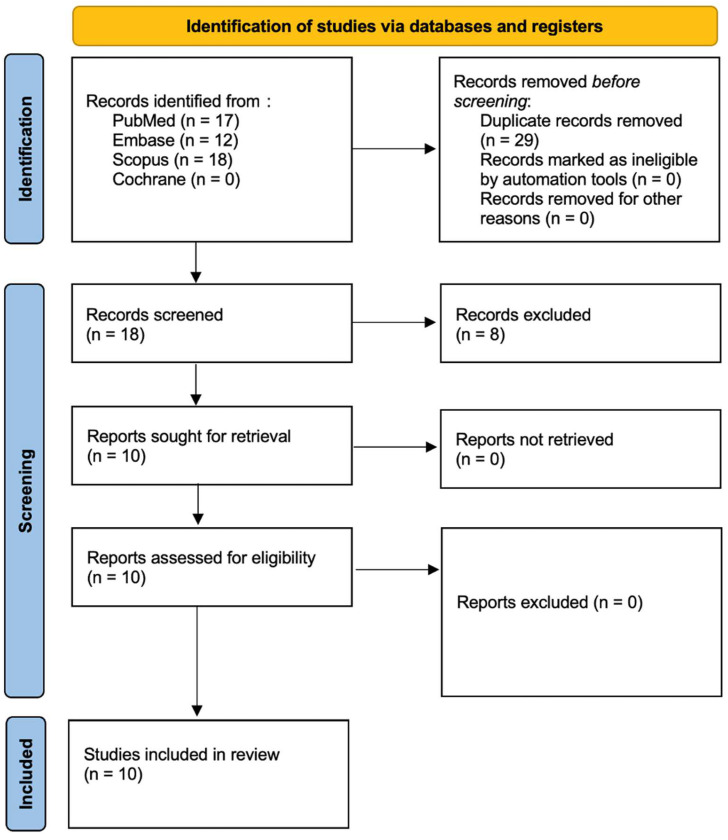
PRISMA 2020 flow diagram.

This systematic review, registered with PROSPERO [30] (ID: CRD420251031368), was designed and executed in accordance with the PRISMA 2020 guidelines for transparent and structured reporting of systematic reviews [31]. A thorough and methodical literature search was conducted across major electronic databases: PubMed/Medline, Embase, Scopus, and the Cochrane Library, to identify relevant studies evaluating the antibacterial and bactericidal effects of the Er:YAG laser. The full search protocol is illustrated in Figure 1. Three independent reviewers carried out database queries using a predefined set of search terms tailored to microbiological outcomes associated with Er:YAG laser application. Language filters were applied to limit inclusion to studies published in English between 1 January 2015 and 3 March 2025. The selection process began with title and abstract screening to assess potential eligibility based on established inclusion criteria (outlined in Table 1), followed by an in-depth full-text review independently conducted by two authors. To enhance the comprehensiveness of the review, a snowballing strategy was also implemented wherein the reference lists of included articles were examined for additional relevant studies. The overarching aim of this review was to synthesize microbiological evidence regarding the efficacy of Er:YAG laser treatment in reducing or eradicating bacterial pathogens, whether as a monotherapy or as an adjunct to conventional antimicrobial approaches. All final inclusions were determined based on rigorous adherence to predefined eligibility parameters.

### 2.3. Study Selection Process

To maintain methodological robustness and minimize potential bias, all identified records were subjected to a rigorous, independent screening process conducted by multiple reviewers. Titles and abstracts were systematically assessed for alignment with the predefined inclusion criteria. In cases of disagreement, reviewers engaged in consensus discussions to resolve discrepancies and ensure consistent decision-making. The selection criteria are as follows:

#### 2.3.1. Inclusion Criteria

- Experimental studies investigating the antimicrobial or bactericidal effects of the Er:YAG laser, conducted either in vitro or in animal models.
- Studies assessing microbial susceptibility to Er:YAG laser treatment were included only if they involved clinically significant oral pathogens (e.g., Gram-positive or Gram-negative bacteria and fungi such as *E. faecalis*, *S. mutans*, *P. gingivalis*, *C. albicans*) relevant to dental infections.
- Investigations exploring potential synergistic effects when Er:YAG laser therapy is combined with conventional antimicrobial agents.
- Studies employing controlled experimental designs, including comparisons with untreated groups, placebo interventions, or other antimicrobial technologies.
- Studies that clearly state the bacteria the Er:YAG laser was tested on.
- Research directly comparing the efficacy of Er:YAG laser treatment with that of standard antimicrobial therapies in terms of microbial load reduction or eradication.
- Studies incorporating follow-up assessments to evaluate the durability of antimicrobial effects and any recurrence of microbial growth post-treatment.

#### 2.3.2. Exclusion Criteria

- Non-scholarly publications, including conference abstracts, case reports, editorials, opinion articles, book chapters, and unpublished theses.
- Studies not published in peer-reviewed journals or lacking sufficient scientific rigor.
- Articles written in languages other than English.
- Redundant publications, such as duplicate reports or multiple articles derived from the same study population without presenting new or distinct data.
- Research unrelated to the treatment of infectious diseases or focused on non-infectious conditions.
- Studies that do not include a comparison or control group to contextualize antimicrobial outcomes.
- Investigations in which the Er:YAG laser is not used as an antimicrobial therapeutic modality.
- Studies using other laser types or technologies without direct evaluation of Er:YAG laser efficacy.
- Research addressing irrelevant pathogens or general microbiological studies without specific outcomes related to bacterial or fungal eradication.
- In vitro studies conducted under highly artificial conditions that limit translational or clinical relevance.

This meticulous, multi-step evaluation, aligned with the PRISMA 2020 guidelines, was designed to uphold transparency and reproducibility [31]. Only studies demonstrating clear relevance and methodological soundness were selected for inclusion. This stringent selection framework was implemented to produce a credible and evidence-based synthesis of current microbiological data on the antibacterial and bactericidal performance of the Er:YAG laser in the context of infection management.

### 2.4. Risk of Bias in Individual Studies

To ensure objectivity and reduce the risk of selection bias during the screening phase, all titles and abstracts identified through the search strategy were independently assessed by multiple reviewers. The level of inter-reviewer agreement was quantified using Cohen’s kappa statistic (κ = 0.82), providing a standardized measure of consistency in decision-making [32]. Discrepancies regarding study inclusion were resolved through structured discussions until a unanimous consensus was achieved. This systematic and collaborative method was employed to uphold the methodological rigor of the review and maintain the integrity of the study selection process.

### 2.5. Quality Assessment

The methodological quality of the included studies was independently assessed by three reviewers, focusing on key elements relevant to the design, execution, and reporting of Er:YAG laser interventions. A structured scoring system was applied to evaluate risk of bias, with each study receiving 1 point for meeting a criterion (“yes”) and 0 points for not meeting it (“no”), based on nine predefined items:

(1) Clear reporting of Er:YAG laser operating parameters (e.g., energy settings, frequency, pulse duration);
(2) Identification of the laser device or manufacturer;
(3) Detailed description of the irradiation protocol, including exposure time and treatment area;
(4) Provision of full technical specifications such as wavelength, spot size, energy fluence, and repetition rate;
(5) Use of dosimetric validation tools such as a power meter;
(6) Inclusion of an appropriate control group (e.g., untreated, placebo, or comparative intervention);
(7) Use of valid statistical analysis for microbiological outcomes;
(8) Transparency in outcome reporting, with no selective or missing data;
(9) Absence of conflicts of interest or undue influence from funding sources.

Each study was assigned a total score out of nine, with risk of bias categorized as high (0–3 points), moderate (4–6 points), or low (7–9 points). Final quality judgments were made in alignment with the guidance provided by the Cochrane Handbook for Systematic Reviews of Interventions [33]. The results of this assessment are presented in Table 2.

### 2.6. Data Extraction

Following consensus on the final selection of studies for inclusion, two reviewers independently performed data extraction using a standardized protocol to ensure consistency and accuracy. Extracted data included bibliographic information (first author, year of publication), study design, microbial species or strains investigated, composition of experimental and control groups, duration of follow-up (if applicable), primary and secondary outcome measures, detailed technical specifications of the Er:YAG laser (e.g., energy settings, pulse duration, wavelength), any adjunctive treatments used, and relevant procedural parameters such as treatment duration and exposure conditions.

## 3. Results

### 3.1. Study Selection

In accordance with the PRISMA 2020 guidelines [31], the study selection process is illustrated in Figure 1. The initial database search yielded 47 records, which were narrowed down to 10 unique studies after duplicate removal. Title and abstract screening confirmed the relevance of these articles, all of which proceeded to full-text review. No studies were excluded at this stage, resulting in a final inclusion of 10 publications spanning the last decade. These studies were deemed suitable for synthesis based on their investigation of the antibacterial and bactericidal efficacy of the Er:YAG laser. A detailed overview of each study’s design, methodology, and outcomes is presented in Table 3. The search identified only in vitro studies. More in vivo studies are needed.

### 3.2. Data Presentation

A comprehensive summary of the findings from the 10 included studies is provided in Table 4, Table 5 and Table 6, offering a structured and accessible presentation of key outcomes, methodological features, and microbiological evidence related to the antibacterial and bactericidal effects of the Er:YAG laser.

### 3.3. Overview of Study Characteristics

Table 4 presents the fundamental characteristics of the studies included in this review, emphasizing variations in experimental design, targeted microbial species, Er:YAG laser treatment parameters, and the criteria used to assess antibacterial and bactericidal outcomes.

### 3.4. Characteristics of Light Sources Used in PDT

Table 6 presents the key physical properties of the light sources utilized in the studies meeting the inclusion criteria.

## 4. Discussion

### 4.1. Results in the Context of Other Evidence

The Er:YAG laser consistently demonstrates significant antibacterial efficacy against a broad range of oral pathogens, including *E. faecalis*, *S. mutans*, *P. gingivalis*, and *C. albicans* [34,35,36,37,38,39,40,41,42,43]. When combined with chemical irrigants or adjunctive agents, it often produces synergistic or additive bactericidal effects, outperforming either modality used alone [34,37,42]. Optimal laser parameters such as energy output, frequency, and pulse duration—are crucial for maximizing bacterial reduction while preserving healthy tissue [38,40,43]. The Er:YAG laser efficiently removes biofilms from titanium surfaces with minimal or no damage, thus maintaining implant structural integrity [36,39,40]. Photoacoustic streaming with the Er:YAG laser enhances disinfection in complex anatomies, including root canals and peri-implant spaces [34,41,43]. Its antibacterial efficacy can be comparable or superior to conventional approaches like ultrasonic irrigation, sodium hypochlorite, or chlorhexidine [34,35,42]. Low-concentration antiseptics combined with the Er:YAG laser have been shown to reduce microbial loads below detectable limits in some protocols [37]. The laser proves effective against both planktonic cells and mature biofilms, highlighting its capacity to eliminate resilient microbial communities [38,40]. Er:YAG treatment can also improve or maintain surface wettability and topography, which is beneficial for subsequent clinical procedures [36,39]. Overall, the Er:YAG laser emerges as a versatile, minimally invasive option that can complement or substitute traditional disinfection methods, though further standardized and clinically oriented studies are required to validate these findings [34,35,41,42,43].

The results of this systematic review corroborate a considerable body of prior investigations, all emphasizing the pronounced bactericidal potency of Er:YAG lasers across endodontic and periodontal applications [39]. Numerous studies offer compelling evidence for this laser’s ability to significantly diminish microbial populations in various clinical contexts, often outperforming alternative modalities or traditional approaches. Sebbane et al. demonstrated that Er:YAG laser irradiation, delivered via a novel side-firing spiral Endo tip, led to a remarkable reduction in *Enterococcus faecalis* biofilm within root canals. The most robust antibacterial impact occurred when 17% EDTA was employed, followed by a final rinse with 2.5% NaOCl [44]. Moritz et al. similarly found that Er:YAG, Nd:YAG, and Ho:YAG lasers significantly decreased bacterial counts in infected root canals; notably, the Er:YAG laser achieved the highest average bacterial eradication rate, registering at 99.64% [45]. Further supporting these findings, Bao et al. reported that Er:YAG laser-activated irrigation methods, including PIPS and SWEEPS, were superior to conventional needle irrigation, passive ultrasonic irrigation, and sonic-powered irrigation for removing multispecies biofilms from both apical artificial grooves and dentinal tubules [46]. Yang et al. observed that supplementing Er:YAG laser treatment with photodynamic therapy substantially enhanced its bactericidal effect against *E. faecalis*, reaching disinfection outcomes comparable to the Er:YAG laser combined with NaOCl [47]. Meanwhile, Ando et al. revealed that the Er:YAG laser exerted a potent bactericidal effect on *Porphyromonas gingivalis* and Actinobacillus actinomycetemcomitans under in vitro conditions, with significant bacterial reduction seen at relatively low energy levels, starting from 0.3 J/cm^2^ [48,49,50,51,52]. Kranendonk et al. further reinforced the laser’s efficacy by showing that Nd:YAG irradiation destroyed all viable cells of six periodontal pathogens within a 15 s exposure, indicating complete bactericidal action in vitro [53]. In addition to these findings, several reports have verified that combining Er:YAG laser irradiation with adjunctive chemical irrigants, such as sodium hypochlorite or hydrogen peroxide, can yield synergistic antibacterial effects—consistent with the present review’s observation that multimodal disinfection regimens frequently surpass monotherapies [44]. This heightened effectiveness appears closely tied to the laser’s photothermal properties, which compromise microbial cell walls and simultaneously promote deeper irrigant penetration into anatomically intricate areas [45].

Investigations into other laser systems, such as Er-, Cr:YSGG and diode lasers, suggest that while these alternatives can achieve clinically meaningful disinfection, Er:YAG lasers frequently deliver superior biofilm disruption and penetration depth, especially in complex clinical scenarios like narrow root canals or around implant surfaces [46]. Such advantages resonate with our findings that the Er:YAG laser can substantially lower microbial loads across a spectrum of pathogens—including *E. faecalis*, *S. mutans*, and *C. albicans*, without causing deleterious effects on tooth or implant structures [47]. Concurrent research on photoacoustic streaming reveals that specific the Er:YAG laser parameter selections can further optimize fluid dynamics, facilitating the removal of biofilms from difficult-to-access regions. These discoveries underscore the critical need to fine-tune energy settings and pulse durations for maximum clinical benefit [48]. Nevertheless, certain inconsistencies in outcomes across different studies may stem from methodological variations, including laser parameters, treatment duration, and the bacterial or fungal strains targeted [49]. Looking ahead, comprehensive in vivo investigations—particularly multicenter randomized controlled trials—are essential for standardizing Er:YAG laser protocols and confirming their meaningful influence on sustained treatment success [50]. As the literature on Er:YAG use continues to expand, it is plausible that clinicians will increasingly incorporate this technology into conventional therapeutic strategies, capitalizing on its robust antibacterial effects, efficient disruption of biofilms, and preservation of healthy tissue [51,52,53]. By integrating these evidence-based insights, the dental community can refine clinical protocols and ultimately enhance patient outcomes in both endodontic and periodontal care.

Laser-activated antibacterial nanoparticles (NPs) have recently emerged as a promising adjunctive strategy for oral bacterial eradication. These nanoparticles—often composed of gold, silver, zinc oxide, titanium dioxide, or graphene-based composites—can be functionalized with photosensitizers or antimicrobial agents and activated by specific laser wavelengths to exert targeted antimicrobial effects [1,2,3]. Upon laser irradiation, these NPs can generate localized heat (photothermal effect), reactive oxygen species (photodynamic effect), or even release embedded bactericidal agents, resulting in enhanced biofilm disruption and microbial death [4,5,6]. This approach offers multiple advantages: improved specificity, deeper penetration into infected tissues, and reduced collateral damage to host cells compared to systemic antimicrobials [7]. In the context of dental infections, laser-activated NPs have shown efficacy against key pathogens such as *Streptococcus mutans*, *Porphyromonas gingivalis*, and *Enterococcus faecalis*, both in planktonic and biofilm states [8,9,10]. Notably, gold and graphene oxide nanoparticles combined with diode or Er:YAG lasers have demonstrated synergistic antibacterial activity, particularly through enhanced photothermal destruction and improved permeability of bacterial membranes [11,12,54,55]. Despite their potential, clinical translation of this technique is still limited due to concerns about long-term biocompatibility, toxicity, and regulatory approval pathways [13,14]. Further studies are needed to optimize NP formulations, laser parameters, and delivery systems to ensure safety and reproducibility in vivo.

### 4.2. Limitations of the Evidence

The current body of research is hindered by substantial variability in both study designs and methodologies, making it difficult to derive clear, universally applicable conclusions. Much of the work has been conducted using in vitro models that do not fully capture the complexity of human biology or real-world conditions, thereby limiting the practical relevance of the outcomes. Furthermore, the lack of standardized protocols—particularly concerning characterization of particles, exposure scenarios, and assessment parameters, leads to inconsistent results that are often impossible to compare directly. Short-term experimental setups further constrain understanding of any long-term impacts, as they offer limited insight into potential chronic or cumulative effects. In addition, restricting the review to English-language publications and omitting the gray literature may introduce a selection bias that excludes potentially significant data. Collectively, these factors emphasize an urgent need for broader, more rigorous, and standardized investigations that encompass long-term observations and more accurately reflect real-life exposures.

### 4.3. Limitations of the Review Process

Despite yielding informative insights, this systematic review faces several inherent limitations. First, the predominance of in vitro experimental designs restricts the direct clinical applicability of the findings, as laboratory conditions do not fully capture the complexities of in vivo environments. While this does not limit the validity of our conclusion, more experimental in vivo evidence must be collected. Second, the studies included exhibit notable heterogeneity in laser parameters, treatment durations, and microbial species, complicating direct comparisons and meta-analyses. Third, the exclusive focus on English-language publications and peer-reviewed articles raises the possibility of publication bias, thereby potentially excluding relevant data reported in other languages or in the gray literature. Additionally, many studies have relatively short follow-up periods, limiting insights into longer-term outcomes and potential microbial recolonization. These methodological and reporting variations underscore the need for more standardized protocols, multicenter trials, and extended observation periods to better validate and generalize the antibacterial efficacy of Er:YAG laser treatment.

### 4.4. Implications for Practice, Policy, and Future Research

Er:YAG laser therapy shows considerable promise for enhancing disinfection in dental procedures, offering a minimally invasive yet highly targeted approach to bacterial and biofilm reduction. Clinically, these findings support the integration of Er:YAG lasers as either a standalone or adjunctive method, particularly in challenging areas such as root canal systems, subgingival regions, and peri-implant spaces. However, one practical concern is the higher upfront cost of laser devices compared to conventional chemical irrigants. This may initially increase the cost of treatment, particularly in practices without existing laser infrastructure. Nevertheless, the long-term sustainability of Er:YAG laser therapy lies in its potential to reduce treatment failures, minimize reinfection, and decrease the need for retreatment, outcomes that could offset the initial investment. Moreover, laser treatment often results in less procedural discomfort and faster healing, potentially enhancing patient satisfaction and reducing chair time. From a sustainability standpoint, the Er:YAG laser offers an eco-friendlier alternative by reducing the volume and disposal burden of chemical irrigants such as sodium hypochlorite and chlorhexidine, which carry cytotoxic and environmental risks. While we do not propose completely replacing chemical agents, laser technology may reduce our dependence on them and mitigate their drawbacks when used in conjunction with low-concentration adjuncts. From a policy perspective, adopting standardized protocols and evidence-based guidelines for Er:YAG laser use could streamline training, ensure consistent treatment outcomes, and foster greater acceptance among practitioners. Additionally, policymakers and professional organizations may consider promoting reimbursement models or financial incentives to encourage broader adoption, given the laser’s potential to improve long-term treatment success and minimize reliance on chemical irrigants. Future research must emphasize well-designed, multicenter clinical trials to confirm and extend current in vitro findings, clarify optimal energy settings, and determine the most effective adjunctive irrigants or antimicrobial agents. Investigations into patient-centered outcomes, cost-effectiveness, and long-term stability of results would further elucidate Er:YAG laser therapy’s role in mainstream dental practice. Rigorous studies examining its impact on microbial resistance, as well as the compatibility of various laser parameters with innovative materials and restoration techniques, will also be crucial in refining this technology for broader, more impactful clinical implementation. Specific unresolved questions that future clinical trials should address include: what are the optimal Er:YAG laser parameters (e.g., energy output, pulse duration, frequency) that maximize bacterial eradication while preserving tissue integrity; how does Er:YAG laser disinfection compare with standard chemical irrigants (e.g., sodium hypochlorite, chlorhexidine) in terms of clinical efficacy, safety, and long-term outcomes; can Er:YAG laser treatment reduce the incidence of reinfection or microbial recolonization in root canal or peri-implant therapy; what is the clinical effectiveness of Er:YAG lasers in challenging anatomical sites, such as curved root canals or peri-implant defects; what are the patient-centered outcomes (e.g., post-operative discomfort, healing time, satisfaction) associated with Er:YAG laser use compared to conventional treatments; and are there cost-effectiveness or workflow benefits that support broader clinical adoption of laser-based disinfection.

## 5. Conclusions

This review confirms the in vitro antibacterial efficacy of the Er:YAG laser against key oral pathogens, including *E. faecalis*, *S. mutans*, *P. gingivalis*, and *C. albicans*, particularly in complex anatomical sites such as root canals and peri-implant regions. The laser’s ability to disrupt biofilms while preserving the integrity of dentin, enamel, and titanium supports its value in minimally invasive decontamination. To enable clinical adoption, future research should focus on in vivo trials, parameter standardization, and comparative studies with conventional methods. Investigating synergies with irrigants or photodynamic agents, as well as patient-centered outcomes, will be essential to guide evidence-based use. Looking ahead, the Er:YAG laser holds promise for broader use in conservative endodontics, peri-implant care, and periodontal therapy, especially when integrated with emerging techniques like nanoparticle-assisted disinfection. While diode lasers may offer a cost-effective alternative for soft tissue decontamination, their lower wavelength and lack of hard tissue interaction limit their use in root canal or mineralized tissue procedures. Diodes are unlikely to replace Er:YAG lasers but may serve as complementary tools, selected based on target tissue and treatment goals. In conclusion, the Er:YAG laser represents a versatile and effective antibacterial tool with expanding potential in dental practice. Continued high-quality research is needed to define its optimal clinical role and integrate it safely into routine protocols.

## Figures and Tables

**Table 1:** Search syntax used in the study.

Source	Search Term	Filters	Number of Results
PubMed	(“Er:YAG laser”[Title/Abstract] OR “erbium:YAG laser”[Title/Abstract]) AND (disinfection[Title/Abstract] OR antibacterial[Title/Abstract] OR bactericidal[Title/Abstract]) AND (efficacy[Title/Abstract] OR effectiveness[Title/Abstract]) AND (bacteria[Title/Abstract] OR microbial[Title/Abstract] OR microbiological[Title/Abstract])	English language Publication years: 2015–2025 Full text	17
Embase	(‘er:yag laser’:ti,ab OR ‘erbium:yag laser’:ti,ab) AND (disinfection:ti,ab OR antibacterial:ti,ab OR bactericidal:ti,ab) AND (efficacy:ti,ab OR effectiveness:ti,ab) AND (bacteria:ti,ab OR microbial:ti,ab OR microbiological:ti,ab)	English language Publication years: 2015–2025	12
Scopus	(TITLE-ABS(“Er:YAG laser”) OR TITLE-ABS(“erbium:YAG laser”)) AND (TITLE-ABS(disinfection) OR TITLE-ABS(antibacterial) OR TITLE-ABS(bactericidal)) AND (TITLE-ABS(efficacy) OR TITLE-ABS(effectiveness)) AND (TITLE-ABS(bacteria) OR TITLE-ABS(microbial) OR TITLE-ABS(microbiological))	English language Publication years: 2015–2025	18
Cochrane	(“Er:YAG laser”:ti,ab OR “erbium:YAG laser”:ti,ab) AND (disinfection:ti,ab OR antibacterial:ti,ab OR bactericidal:ti,ab) AND (efficacy:ti,ab OR effectiveness:ti,ab) AND (bacteria:ti,ab OR microbial:ti,ab OR microbiological:ti,ab)	English language Publication years: 2015–2025	0

**Table 2:** The results of the quality assessment and risk of bias across the studies.

Study	1	2	3	4	5	6	7	8	9	Total	Risk
Shan et al., 2022 [34]	1	0	1	1	0	1	1	1	1	7	Low
Alzahrani et al., 2022 [35]	0	0	0	0	0	1	1	1	1	4	Moderate
Amid et al., 2021 [36]	1	0	1	1	0	1	1	1	1	7	Low
Deeb et al., 2019 [37]	1	0	1	1	0	1	1	1	1	7	Low
Grzech-Leśniak et al., 2025 [38]	1	1	1	1	0	1	1	1	1	8	Low
Homayouni et al., 2019 [39]	1	0	1	1	0	1	1	1	1	7	Low
Polak et al., 2020 [40]	1	0	1	1	0	1	1	1	1	7	Low
Seghayer et al., 2023 [41]	1	0	1	1	0	1	1	1	1	7	Low
Chohan et al., 2024 [42]	1	0	1	1	0	1	1	1	1	7	Low
Terlep et al., 2023 [43]	1	1	1	1	0	1	1	1	0	7	Low

**Table 3:** A general overview of the studies.

Study	Country	Study Type
Shan et al., 2022 [34]	China	In vitro
Alzahrani et al., 2022 [35]	Saudi Arabia	In vitro
Amid et al., 2021 [36]	Iran	In vitro
Deeb et al., 2019 [37]	USA/Poland	In vitro
Grzech-Leśniak et al., 2025 [38]	Poland	In vitro
Homayouni et al., 2019 [39]	Iran	In vitro
Polak et al., 2020 [40]	Israel	In vitro
Seghayer et al., 2023 [41]	Hong Kong	In vitro
Chohan et al., 2024 [42]	India	In vitro
Terlep et al., 2023 [43]	Slovenia	In vitro

**Table 4:** A summary of the molecular aspects of the Er:YAG laser.

Property	Details
Wavelength	2940 nm (mid-infrared)
Absorption Characteristics	Highly absorbed by water and hydroxyapatite, enabling precise ablation of dental hard tissues
Primary Mechanism	Laser-induced ablation through rapid vaporization of water; removes smear layer and opens dentinal tubules
Photophysical Effects	- Cavitation effect (bubble expansion and collapse) - Shock wave generation - Swing washing effect - Moses effect (enhanced fluid dynamics)
Impact on Irrigant Penetration	Promotes deep penetration of irrigants (up to 961.5 μm in dentinal tubules), enhancing antimicrobial action
Smear Layer and Biofilm Removal	Effectively removes smear layer and biofilms, exposing intratubular bacteria to irrigants
Bactericidal Efficacy	Effective against *E. faecalis* in root canal and dentinal tubules
Thermal and Morphological Safety	Minimal thermal damage and morphological alteration to surrounding periodontal tissue
Operational Application	Non-contact; fiber tip positioned in pulp chamber (not within canal), making it suitable for minimally invasive access
Comparison with Ultrasonic	Similar or better antibacterial efficacy; superior in deep dentin disinfection; safer and simpler to operate

Source: [34,35,36,37,38,39,40,41,42,43].

**Table 5:** Main outcomes and details from each study.

Author and Year	Cells Evaluated	Study Groups	Outcomes
Shan et al., 2022 [34]	*Enterococcus faecalis (ATCC 29212)*	Sixty-six extracted maxillary first molars were divided into two main groups based on the access cavity design: conventionally invasive access (CIA/group 1) and computer-guided minimally invasive access (MIA/group 2). Each of these was further subdivided into three experimental subgroups (*n* = 9 per subgroup) depending on the irrigation method: CI, PUI, and LAI. Thus, six experimental groups were formed: 1A, 1B, 1C, 2A, 2B, and 2C.	- Both PUI and LAI significantly reduced bacterial CFU counts compared to CI in both CIA and MIA groups. - Er:YAG laser achieved the lowest bacterial counts in dentinal tubules, indicating superior penetration and disinfection. - CI under MIA (Group 2A) left significantly more residual bacteria than under CIA (Group 1A). - No significant difference in antibacterial efficacy was observed between access types for PUI and LAI. - Er:YAG laser showed superior antibacterial efficacy and was easier to use in conservative access cavities than PUI.
Alzahrani et al., 2022 [35]	*Candida albicans (ATCC 10231)*, *Staphylococcus aureus (ATCC 25923) Streptococcus mutans (ATCC 25175)*, *Escherichia coli (ATCC 25922)*	Fifty PMMA DBP samples were fabricated and randomly divided into five groups (*n* = 10) based on the disinfection method used. Group 1 was treated with the Er:YAG laser; Group 2 with RB photoactivated by a red LED; Group 3 with autoclaving; Group 4 with 0.12% CHX, serving as the control; and Group 5 with chitosan, photoactivated by a diode laser. Each group underwent microbial contamination followed by disinfection, and both antimicrobial efficacy and fracture strength of the DBP were assessed.	- Er:YAG laser, chitosan, autoclave, and CHX showed significant and comparable antimicrobial effects. - Rose Bengal was significantly less effective against *C. albicans* and *S. aureus*. - CHX had the highest antimicrobial efficacy and fracture resistance. - Er:YAG laser and chitosan matched CHX in microbial reduction and preserved fracture strength—viable CHX alternatives. - Autoclave was effective in disinfection but significantly weakened DBP fracture strength.
Amid et al., 2021 [36]	*Escherichia coli (ATCC 25922)*	A total of 28 titanium disks were included in the study. Of these, 24 were contaminated with E. coli and subsequently randomized into three groups: a positive control group (contaminated, untreated), a laser treatment group (Er:YAG laser, 150 mJ/pulse at 10 Hz), and an air-flow abrasion group using glycine powder. Four discs served as the negative control (uncontaminated, untreated). Each treated group underwent decontamination followed by immersion in a hydrogen peroxide and silver salt solution to simulate clinical rinsing conditions.	- Both Er:YAG laser and air-flow abrasion significantly improved wettability by reducing the contact angle on titanium surfaces. - Air-flow abrasion achieved the lowest contact angle, but not significantly different from the laser group. - Elemental analysis showed air-flow abrasion had the most favorable surface composition: lower carbon and higher titanium levels vs. positive control. - SEM showed no major surface damage in either group; air-flow surfaces resembled the negative control. - Air-flow abrasion provided superior decontamination without surface damage; Er:YAG laser was effective but showed slight chemical differences.
Deeb et al., 2019 [37]	*Streptococcus gordonii (ATCC 10558)*, *Fusobacterium nucleatum (ATCC 25566)*, *Porphyromonas gingivalis (W83)*	Eight treatment groups were established for each bacterial species: (1) untreated control, (2) 0.5% H_2_O_2_, (3) 0.5% NaOCl, (4) 0.03% chlorhexidine (CHX), (5) Er:YAG laser alone, (6) Er:YAG + 0.5% H_2_O_2_, (7) Er:YAG + 0.5% NaOCl, and (8) Er:YAG + 0.03% CHX. The Er:YAG laser was applied using clinical periodontal settings (40 mJ, 40 Hz, 1.6 W for 20 s) with a 400-μm Varian fiber tip in contact mode. Each experiment was conducted in duplicate and repeated on four separate days to ensure reliability.	- Er:YAG laser alone had limited, non-significant antibacterial effects. Most effective monotherapies: 0.5% H_2_O_2_ and 0.5% NaOCl (*p* < 0.001), 0.03% CHX showed moderate effect, especially against *F. nucleatum*. - Combination treatments (Er:YAG + irrigant) yielded the highest bacterial reduction. - For *S. gordonii*: all combinations significantly outperformed monotherapies (*p* < 0.05). - For *P. gingivalis*: only Er:YAG + CHX improved results over CHX alone (*p* < 0.01). - For *F. nucleatum*: Er:YAG + H_2_O_2_ or NaOCl reduced bacteria below detectable limit. - Combining Er:YAG with dilute antiseptics enhances bactericidal efficacy beyond individual treatments.
Grzech-Leśniak et al., 2025 [38]	*Candida albicans*, *Candida glabrata*, and *Streptococcus mutans (Clinical strains)*	The experiment included control (no irradiation) and two test groups subjected to different Er:YAG laser settings: T1 (low power, 0.15 W, 2 Hz, 11.3 W/cm^2^) and T2 (higher power, 1.6 W, 40 Hz, 120.54 W/cm^2^). Both planktonic cultures and biofilms—single-species and dual-species (e.g., *C*. *albicans* + *S. mutans*, *C. glabrata* + *S. mutans*, *C. albicans* + *C. glabrata*)—were prepared and treated under these laser conditions to assess microbial viability through CFU counts and crystal violet assays for biomass.	- Er:YAG laser significantly reduced microbial counts in both planktonic and biofilm cultures. - T2 setting (higher irradiance) achieved >99.9% CFU reduction in dual-species biofilms of *C. albicans* and *S. mutans*. - For *C. glabrata*, T1 (lower setting) was more effective in some dual-species contexts. - T1 better reduced biofilm biomass, especially in *C. albicans*–*S. mutans* and *C. glabrata*–*S. mutans* combinations. - Optimal laser settings depend on microbial composition, underscoring the need for tailored Er:YAG protocols for complex oral biofilms.
Homayouni et al., 2019 [39]	*Micrococcus luteus (ATCC 10240)*, *Acinetobacter baumannii (ATCC 19606)*, *Enterococcus faecalis (ATCC 29212)*, *Candida albicans (ATCC 10231)*, *and Bacillus subtilis (ATCC 6633)*	Titanium disks were divided into six decontamination groups: (1) high-pressure steam (4 MPa for 5 s), (2) 1% NaOCl, (3) 3% H_2_O_2_, (4) GaAlAs diode laser (810 nm, 1 W), (5) Er:YAG laser (2940 nm, 100 mJ, 10 Hz), and (6) an untreated control. These methods were applied to both the multispecies non–spore-forming group and the *Bacillus subtilis* group. Additional tests were conducted using higher concentrations or power settings to assess the impact on surface roughness.	- All methods significantly reduced microbial load. - Complete elimination of all organisms was achieved with NaOCl, H_2_O_2_, high-pressure steam, and Er:YAG laser. - GaAlAs diode laser reduced >90% of microbes but fully eliminated only *C. albicans*. - NaOCl, especially at higher concentrations, significantly increased surface roughness (Sa, Sq, Sdr). - Er:YAG laser and other methods did not alter surface topography significantly. - Conclusion: Er:YAG laser is an effective, non-destructive decontamination method for titanium abutments in intraoral use.
Polak et al., 2020 [40]	*Fusobacterium nucleatum (ATCC 25586)*, *Porphyromonas gingivalis (ATCC 33277)*, *Streptococcus sanguinis (ATCC 10556)*, *Actinomyces naeslundii (ATCC 17233).*	Titanium disks with established multispecies biofilms were divided into several treatment groups: ultrasonic scaling, hand curets, nylon hand brush, and Er:YAG laser treatment with varied parameters (pulse energies of 20/40/50 mJ, frequencies of 40/45/50 Hz, and tip-to-target distances of 1/3/5 mm). Each group included three replicates. An additional group using the Er:YAG laser handpiece without beam emission (simulating water irrigation only) served to assess the independent effect of water spray. The efficacy of these decontamination methods was evaluated using fluorescent live/dead bacterial staining and microscopy.	- Er:YAG laser was the most effective method for biofilm removal from titanium surfaces. - All laser settings (energy, frequency, tip distance) significantly reduced biofilm with minimal viable bacteria. - Hand curets and ultrasonic devices only partially removed biofilm. - Nylon brushes caused smearing and redistribution of biofilm. - Bactericidal effect attributed to laser light combined with water spray; water spray alone had minimal impact. - All Er:YAG settings showed similar efficacy, allowing flexible clinical application without compromising results.
Seghayer et al., 2023 [41]	*Enterococcus faecalis* (clinical samples)	The 68 infected samples were randomly divided into five groups: (i) PUI (3% NaOCl) (*n* = 16), (ii) Er,Cr:YSGG laser (WTL) with saline (*n* = 16), (iii) Photon-Induced Photoacoustic Streaming (PIPS) with 3% NaOCl (*n* = 16), (iv) positive control group (PC) with infection but no irrigation (n = 10), and (v) NC with no infection and no treatment (*n* = 10). All groups underwent microbiological analysis using both sampling methods to compare the effectiveness of each disinfection protocol.	- PUI and PIPS significantly reduced bacterial loads in the main canal vs. WTL (99.89% and 99.98% vs. 92.06%, paper-point method). - No significant differences among PUI, PIPS, and WTL in the apical 5 mm (pulverization method). - Negative culture counts: - PUI: 8 (paper-point), 10 (pulverization) - PIPS: 7 (both methods) - WTL: 0 (paper-point), 6 (pulverization) - PUI and PIPS appear more effective than WTL in the main canal but not definitively superior in the apical third.
Chohan et al., 2024 [42]	*Enterococcus faecalis*, *Pseudomonas aeruginosa*, and *Candida albicans*	Four disinfection protocols were compared in standardized root canal models: (1) Er:YAG laser therapy applied for 3 min, (2) 5.25% NaOCl irrigation for 5 min, (3) ozone therapy administered for 4 min, and (4) PDT using methylene blue as a photosensitizer activated with a 660 nm diode laser for 9 min. Each treatment was applied to 10 specimens per microbial species, making it a total of 120 treated samples.	- Er:YAG laser showed the highest antimicrobial efficacy against *E. faecalis* and *P. aeruginosa*, followed by PDT. - NaOCl and ozone were less effective in comparison. - No significant differences in efficacy against *Candida albicans* across treatments. - Er:YAG laser achieved the deepest penetration (1.25 mm). - PDT had the longest treatment time (9 min). - Er:YAG laser and PDT are promising alternatives or adjuncts to conventional disinfection, especially for resistant bacteria.
Terlep et al., 2023 [43]	*Enterococcus faecalis*	Two Er:YAG laser photoacoustic irrigation modalities were compared: the single-pulse SSP and the dual-pulse AutoSWEEPS. These were tested in two narrow irrigation model geometries mimicking clinical peri-implant spaces—a square gap and a cylindrical tube. Each modality was applied for two treatment durations (10 s and 60 s), and effects were evaluated on both overall bacterial surface density and live/dead bacterial ratios. No chemical disinfectants were used—only saline solution served as the irrigant medium.	- Both SSP and AutoSWEEPS significantly reduced biofilm surface density. - AutoSWEEPS outperformed SSP, especially at 60 s. - In square gaps: - AutoSWEEPS removed up to 98.5% of biofilm - SSP removed up to 95% - In cylindrical gaps: - AutoSWEEPS: 95% removal - SSP: 93% removal - AutoSWEEPS consistently left fewer viable bacteria, showing superior antimicrobial efficacy. - Dual-pulse AutoSWEEPS is a promising method for cleaning complex, narrow anatomical spaces in peri-implant diseases.

Abbreviations: CIA—Conventionally Invasive Access, MIA—Minimally Invasive Access, CI—Conventional Irrigation, PUI—Passive Ultrasonic Irrigation, LAI—Laser-Activated Irrigation, Er:YAG—Erbium-doped Yttrium Aluminum Garnet, PMMA—Polymethylmethacrylate, DBP—Denture Base Plate, RB—Rose Bengal, CHX—Chlorhexidine, ATCC—American Type Culture Collection, H_2_O_2_—Hydrogen Peroxide, NaOCl—Sodium Hypochlorite, GaAlAs—Gallium Aluminum Arsenide, SEM—Scanning Electron Microscopy, Sa—Arithmetical Mean Height, Sq—Root Mean Square Height, Sdr—Developed Interfacial Area Ratio, WTL—Waterlase (Er,Cr:YSGG Laser), PIPS—Photon-Induced Photoacoustic Streaming, PC—Positive Control, NC—Negative Control, PDT—Photodynamic Therapy, SSP—Super Short Pulse.

**Table 6:** Characteristics of lights sources used.

Author and Year	Light Source	Operating Mode	Energy Density (Fluence) (J/cm^2^)	Power Output (mW)	Irradiation Time (s)
Shan et al., 2022 [34]	Er:YAG laser (Fotona LightWalker, Fotona, Slovenia)	Super Short Pulse		300	60
Alzahrani et al., 2022 [35]	Er:YAG laser (QTS-F04, Beijing, China)	continuous pulsed mode	18.9	1200	150
Amid et al., 2021 [36]	Er:YAG laser (DEKA, Italy)	continuous pulsed mode		1500	30
Deeb et al., 2019 [37]	Er:YAG laser (LightWalker, Fotona, Slovenia)	Short pulse (300 µs pulse duration), contact mode		1600	20
Grzech-Leśniak et al., 2025 [38]	Er:YAG laser (Fotona LightWalker)	Pulsed (300 µs pulse duration)	T1: 5.65 T2: 3.01	T1: 5.65 J/cm^2^ T2: 3.01 J/cm^2^	30
Homayouni et al., 2019 [39]	Er:YAG laser (DEKA Dental Laser Systems, Florence, Italy)	Pulsed mode		1000	60
Polak et al., 2020 [40]	Er:YAG laser (LiteTouch, Light Instruments, Yokneam, Israel)	Pulsed		900	10
Seghayer et al., 2023 [41]	Er,Cr:YSGG laser (Waterlase MD, Biolase, USA) Er:YAG laser (AT Fidelis, Fotona, Slovenia)	H mode (pulsed) Pulsed, 50 µs pulse duration		750 300	180 90 s (3 × 30 s activation cycles)
Chohan et al., 2024 [42]	Er:YAG laser	Pulsed mode			180
Terlep et al., 2023 [43]	Er:YAG laser (LightWalker, Fotona)	SSP (Super Short Pulse), 50 µs pulse duration AutoSWEEPS—dual pulses (2 × 25 µs), variable delay between pulses		300 600	10 or 60 s

## Data Availability

No new data were created or analyzed in this study. Data sharing is not applicable to this article.
